# Performance of Different Diagnostic PD-L1 Clones in Head and Neck Squamous Cell Carcinoma

**DOI:** 10.3389/fmed.2021.640515

**Published:** 2021-04-27

**Authors:** Julika Ribbat-Idel, Franz F. Dressler, Rosemarie Krupar, Christian Watermann, Finn-Ole Paulsen, Patrick Kuppler, Luise Klapper, Anne Offermann, Barbara Wollenberg, Dirk Rades, Simon Laban, Markus Reischl, Karl-Ludwig Bruchhage, Christian Idel, Sven Perner

**Affiliations:** ^1^Institute of Pathology, University of Luebeck and University Hospital Schleswig-Holstein, Luebeck, Germany; ^2^Pathology, Research Center Borstel, Leibniz Lung Center, Borstel, Germany; ^3^Department of Otorhinolaryngology, MRI Technical University Munich, Munich, Germany; ^4^Department of Radiation Oncology, University of Luebeck, Luebeck, Germany; ^5^Department of Otorhinolaryngology, Head and Neck Surgery, University of Ulm, Ulm, Germany; ^6^Karlsruhe Institute of Technology, Institute for Automation and Applied Informatics, Eggenstein-Leopoldshafen, Germany; ^7^Department of Otorhinolaryngology, University of Luebeck, Luebeck, Germany

**Keywords:** HNSCC, PD-L1, checkpoint inhibitors, TMA, harmonization, therapy prediction, protein quantitation

## Abstract

**Background:** The approval of immune checkpoint inhibitors in combination with specific diagnostic biomarkers presents new challenges to pathologists as tumor tissue needs to be tested for expression of programmed death-ligand 1 (PD-L1) for a variety of indications. As there is currently no requirement to use companion diagnostic assays for PD-L1 testing in Germany different clones are used in daily routine. While the correlation of staining results has been tested in various entities, there is no data for head and neck squamous cell carcinomas (HNSCC) so far.

**Methods:** We tested five different PD-L1 clones (SP263, SP142, E1L3N, 22-8, 22C3) on primary HNSCC tumor tissue of 75 patients in the form of tissue microarrays. Stainings of both immune and tumor cells were then assessed and quantified by pathologists to simulate real-world routine diagnostics. The results were analyzed descriptively and the resulting staining pattern across patients was further investigated by principal component analysis and non-negative matrix factorization clustering.

**Results:** Percentages of positive immune and tumor cells varied greatly. Both the resulting combined positive score as well as the eligibility for certain checkpoint inhibitor regimens was therefore strongly dependent on the choice of the antibody. No relevant co-clustering and low similarity of relative staining patterns across patients was found for the different antibodies.

**Conclusions:** Performance of different diagnostic anti PD-L1 antibody clones in HNSCC is less robust and interchangeable compared to reported data from other tumor entities. Determination of PD-L1 expression is critical for therapeutic decision making and may be aided by back-to-back testing of different PD-L1 clones.

## Introduction

In a growing number of solid tumors, immunotherapy by checkpoint blockage targeting programmed death receptor 1 (PD-1) or its ligand programmed death-ligand 1 (PD-L1) has become a standard treatment (1–9). The response to the administration of most PD-1 or PD-L1 inhibitors often correlates with PD-L1 expression determined before therapy using specific companion diagnostics for immunohistochemistry (IHC) resulting in approval in combination with PD-L1 expression as a diagnostic biomarker (8, 9). In HNSCC, this does not hold good for nivolumab which is approved for administration after cisplatin failure, regardless of PD-L1 status determined by IHC. For pembrolizumab, though, it is still necessary to specify PD-L1 status by IHC. Interestingly, most tumor boards still request PD-L1 status from the pathologists to decide for or against a checkpoint inhibitor therapy regime. While IHC is a routine tool in modern pathology to investigate diagnostic and predictive markers in tissue samples, its use in PD-L1 expression analysis has raised several issues: (a) There are different PD-L1 assays each specific to a therapeutic antibody without a common standard. (b) Different scoring systems are applied to different tumor types and indications. (c) Problems of tumor heterogeneity, inter-institutional preanalytics, and inter-/intra-observer variability are being addressed but are difficult to solve (10). Several attempts have been made to compare commercially available PD-L1 clones (11–14). For example, clone 22C3 (Agilent Dako Omnis, Santa Clara, CA, USA) showed high concordance with SP263 (Ventana Medical Systems Roche, Oro Valley, AZ, USA). For non-small cell lung cancer (NSCLC), it was therefore recently CE-marked for interchangeable use with 22C3 and 28-8 (Abcam, Cambridge, UK) (10). Some pathology laboratories use less expensive PD-L1 clones such as 22C3 and E1L3N for different reasons. Like many hospitals, they are dedicated to the economically efficient use of reagents and resources. Similarly, some lack access to the specific immunostainer platform that is necessary to carry out clone-specific stains (e.g., Ventana Benchmark immunostainer for SP263, both Ventana Roche), and there are no commercially available ready to use kits for PD-L1 clones for the widely used Leica Bond platform (Leica Biosystems, Wetzlar, Germany) (10).

Checkpoint and PD-L1 inhibitors like pembrolizumab, atezolizumab, and durvalumab have been approved for first or second-line treatment of PD-L1 positive advanced cancers [e.g., NSCLC (2, 3, 15)], urothelial cancer (16), and triple-negative breast cancer (17). Since then, pathologists are required to report the PD-L1 status, which comprises one or more PD-L1 scores depending on the tumor entity. The tumor positivity score (TPS) is defined as the estimated percentage of tumor cells showing partial or complete membrane staining for PD-L1. It was developed for clone 22C3 as a biomarker for pembrolizumab (9, 18). The immune cell score (IC) is based on the estimated area of PD-L1 positive tumor immune cells in relation to all tumor immune cells. It was developed for SP142 in urothelial carcinoma, NSCLC (15), and TNBC. The combined positivity score (CPS) is supposed to reflect both tumor cell and immune cell PD-L1 expression. It was also developed as a biomarker for pembrolizumab.

In the USA, the Federal Drug Administration (FDA) restricted the application of pembrolizumab for advanced NSCLC to those patients whose tumor samples had been tested using the DAKO 22C3 pharmDx assay (the so-called companion diagnostic assay) (18). In Europe though, the DAKO platform is not as widely used, and the European Medicines Agency (EMA) does not demand a mandatory specific PD-L1 IHC platform or clone (10).

For recurrent HNSCC, two therapeutic anti-PD-1 antibodies are currently used: Nivolumab after cisplatin failure without PD-L1 expression as a biomarker and Pembrolizumab after cisplatin failure and with a TPS ≥50%. Pembrolizumab is also approved for palliative first-line treatment of HNSCC with a CPS≥1 with or without platinum-based chemotherapy. Thus, targeting the same molecule, Pembrolizumab requires PD-L1 expression to be demonstrated by IHC, while the use of Nivolumab is not restricted.

## Materials and Methods

### Tumor Material and Patient Data

The study was conducted according the Declaration of Helsinki. Approval by the University of Luebeck Ethics Committee was obtained (project code AZ 16-277). Tissue samples were routinely fixated in 4% buffered neutral formalin for 12–24 h. After paraffin embedding the preserved tissue blocks were stored at room temperature in our archives until they were retrieved. We accessed our large and comprehensively clinicopathologically characterized HNSCC cohort, as described before (19, 20). For the study at hand, we selected those TMAs from our cohort that contained tumor tissue of recurrent disease and matching primary tumors (*n* = 75 patients). For patient details please refer to Supplementary Table 1.

### TMA Construction

**Figure 1:**
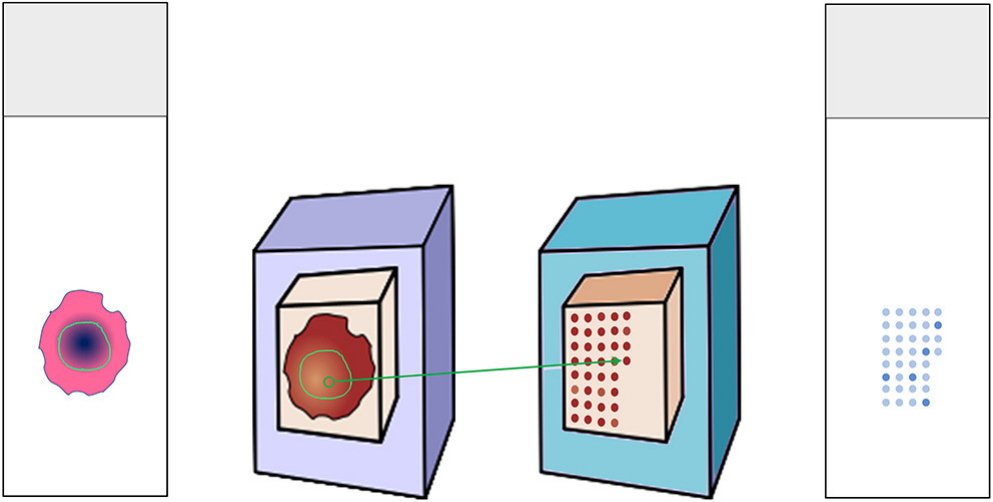
Construction of tissue microarray. Donor H&E slides were annotated for the tumor region. The matching donor blocks were identified and annotated as well. Three cores from the region of interest were punched out and embedded in the recipient paraffin block. The recipient block could then serve for multiple analyses, e.g., immunohistochemical stainings.

H&E slides were annotated for regions of interest (ROI) containing representative squamous cell carcinoma areas. Corresponding paraffin blocks were matched. Manual Tissue Arrayer 1 (Estigen AlphaMetrix Biotech, Rödermark, Germany) was used to construct TMAs as seen in Figure 1. Briefly, 2 mm diameter cores were punched out of the donor block's ROI and embedded into a paraffin recipient block. This was repeated three times for each tumor sample resulting in three cores per patient. One recipient block holds up to 60 triplets. Whenever possible, meaning whenever enough representative tumor tissue could be yielded, we created two more replicas of each TMA.

### Immunohistochemistry and Evaluation of Stains

3 μm thin slices were cut from the TMA recipients' blocks and put on glass slides (Figure 1). All immunohistochemical stainings were performed on a Ventana BenchMark automated staining system (Roche, Basel, Switzerland), as previously described (21). Deparaffinization protocol according to EZ Prep was followed by heat-mediated antigen retrieval (pH 8.4 buffer for up to 32 min; both Ventana Medical Systems Roche, Oro Valley, AZ, USA). Primary antibody was titrated and incubated as follows.

- SP263: incubation for 20 min at 36° (rabbit monoclonal antibody with OptiView DAB IHC Detection Kit, both Ventana Medical Systems Roche)
- SP142: incubation for 8 min at 37° (rabbit monoclonal antibody with OptiView DAB IHC Detection Kit, both Ventana Medical Systems Roche)
- E1L3N: incubation for 60 min at 36° (rabbit monoclonal antibody, RTU, Cell Signaling, Danvers, MA, USA). Counterstaining with haematoxylin (Ventana Medical Systems, Tucson, AZ, USA) and the proper alkalinity was ensured by washing with Bluing reagent, pH = 8.0.
- 28-8: incubation for 40 minutes at 37° (ab205921, rabbit monoclonal antibody, Abcam, Cambridge, UK with OptiView IHC Detection Kit, Ventana Medical Systems Roche)
- 22C3: incubation for 40 min at 37° (mouse monoclonal antibody, Agilent Dako Omnis, Santa Clara, CA, USA with OptiView DAB IHC Detection Kit, Ventana Medical Systems Roche)

Tonsil tissue was employed as positive control (Supplementary Figure 1). PD-L1 IHC stains were then evaluated by two independent pathologists. Tumors were represented by three cores to address tumor heterogeneity. PD-L1 scores were reported separately for each core. Mean values of the three cores were calculated and used as input for score calculation. The resulting expression data contained the number and area shares of tumor and immune cells with the expression as well as the ratio of tumor to immune cell number [necessary to calculate the combined positive score (CPS)]. The latter was calculated as the number of positive immune and tumor cells divided by the number of viable tumor cells, multiplied by and capped at 100. The total positive score (TPS) was calculated as the percentage of tumor cells with positive membrane staining, regardless of staining intensity and continuance. The immune cell score (IC) is the percentage of tumor area occupied by tumor immune cells.

### Statistical Analysis and Visualization

All statistical analyses were performed by custom scripts in Python 2.7 (Enthought, Austin, USA, Canopy distribution 1.1.0.1371) including the scipy, numpy, sklearn, matplotlib, seaborn and pandas packages. Non-negative matrix factorization was performed based on the nimfa package (22). Briefly, the data were z-score transformed, shifted by the minimum value to turn negative into positive values without changing data patterns, and factorized with random seed, rank ^*^ 100 iterations and 100 runs. The resulting data was further analyzed and visualized with the scipy.cluster.hierarchy module.

Apart from Python visualization packages, we used the following software to create artwork and edit photomicrographs. Inkspace (version 0.92.4, The Inkscape Project c/o Software Freedom Conservancy, Brooklyn, NY, USA, https://inkscape.org/), Krita (version 4.2.8, Stichting Krita Foundation, Deventer, The Netherlands, https://krita.org), GIMP (version 2.10.14, The GIMP Project c/o GNOME Foundation, Orinda, CA, USA, https://www.gimp.org).

## Results

### Tumor Material and Patient Data

As described previously, our cohort is well-representative for HNSCCs and well-reflects the aggressive tumor behavior (19). Briefly, our cohort mirrors the typical characteristics for HNSCC patients: three-quarters were men with smoking and alcohol as common nutritive-toxic pathogens. A little <1/3 were p16 positive. The majority offered advanced stages (UICC III/IV) at first-time diagnosis. About 25% of patients experienced a cancer relapse, half of which passed away within 5 years. The 5-year survival for the whole cohort was about 60%.

### Immunohistochemistry and Evaluation of Stains

**Figure 2:**
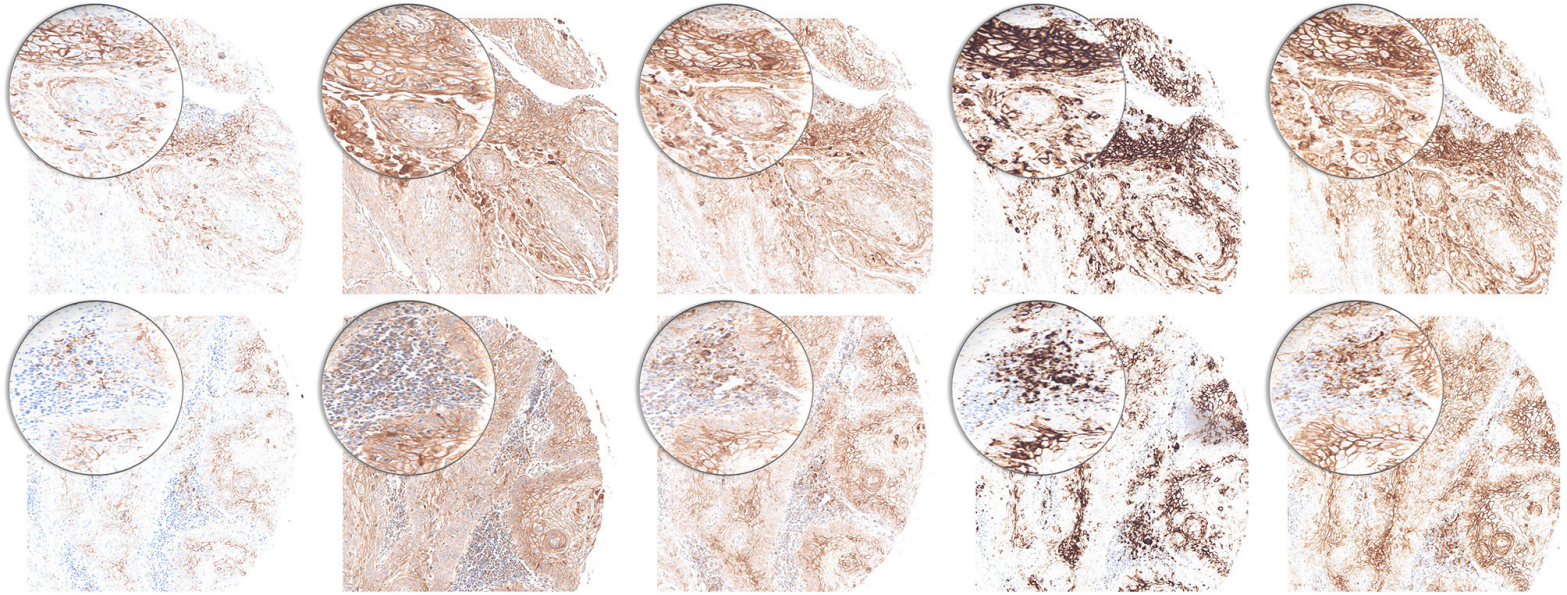
PD-L1 staining pattern in HNSCC. Upper row: tumor cells. Lower row: immune cells. From left to right: 22C3, 28-8, E1L3N, SP142, and SP263. Original magnification x100. Inlay magnification x200.

We performed immunohistochemical PD-L1 stains for five clones (22C3, 28-2, E1L3N, SP142, and SP263) and estimated TPS, IC, and CPS. All five clones delivered satisfactory staining quality with 22C3 being the most discreet. SP142 offered a more grainy aspect. E1L3N, SP263, and 28-8 showed a robust pattern (Figure 2). Positive controls demonstrate staining success (tonsil tissue, Supplementary Figure 1).

### Statistical Analysis

**Figure 3:**
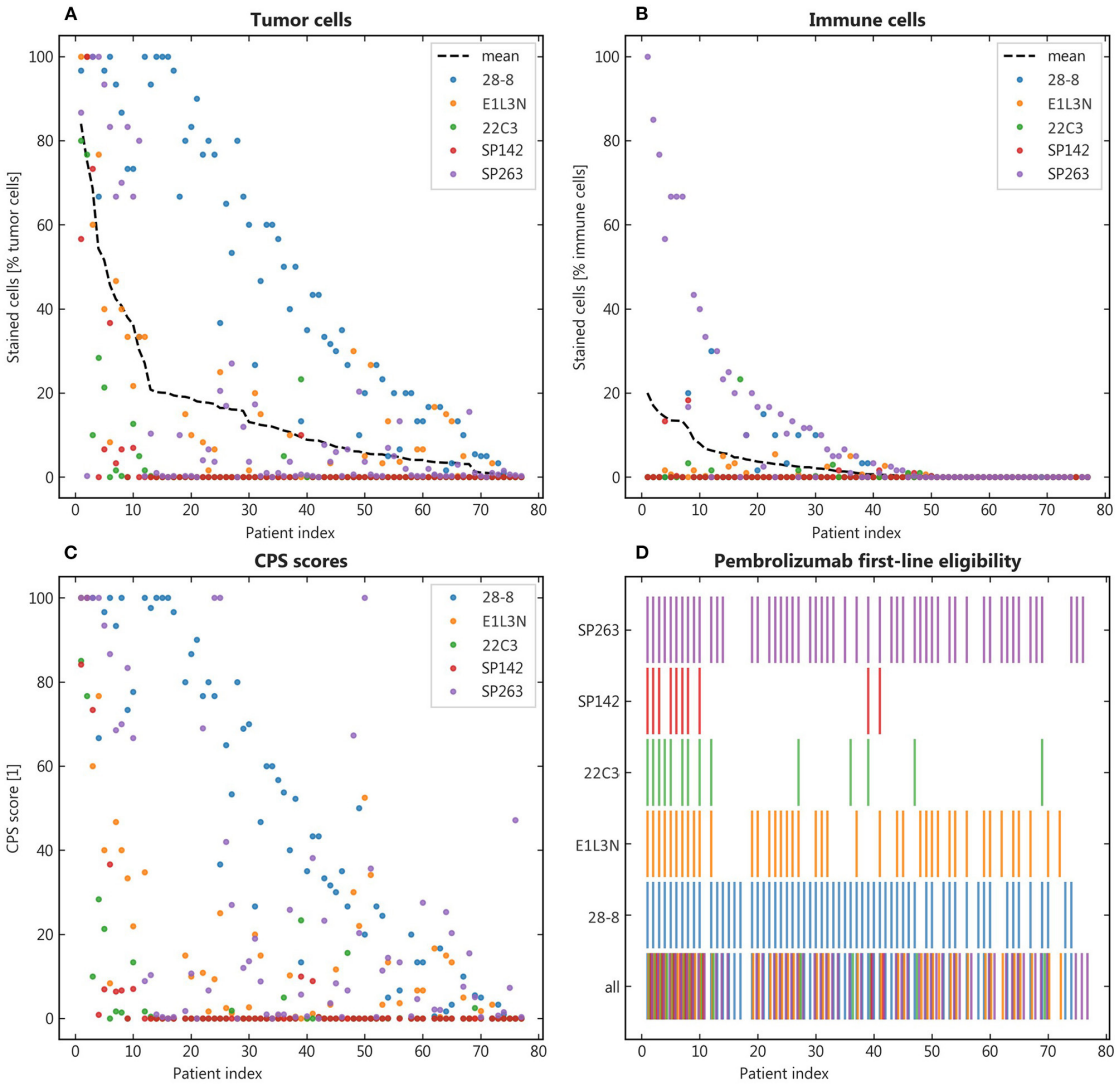
Qualitative results. **(A,B)**: Patients were ranked by their mean positive tumor and immune cells respectively to facilitate comparison across the different antibody clones; **(C)**: The combined positive score (CPS) was calculated for each patient and the resulting eligibility for pembrolizumab portrayed in binary representation (color = eligible) **(D)**.

To illustrate the variety of staining with different clones, the mean share of positive stainings across clones was calculated and the patient samples ranked by this order (Figures 3A,B). The resulting distribution showed marked dispersion, which was also mirrored by the resulting CPS (Figure 3C). As a relevant clinical consequence, the individual eligibility of patients for first-line pembrolizumab was assessed by CPS ≥ 1 (Figure 3D). Thereby, relevant differences between clones were observed with eligible patients ranging from SP142 with 14%, 22C3 with 18% to E1L3N with 48%, SP263 with 68%, and 28-8 with 78% of patients. As other cutoffs are used in clinical trials we also calculated alternative eligibility cutoffs for CPS1, CPS20, CPD50, and TPS50 (Supplementary Figure 2).

**Figure 4:**
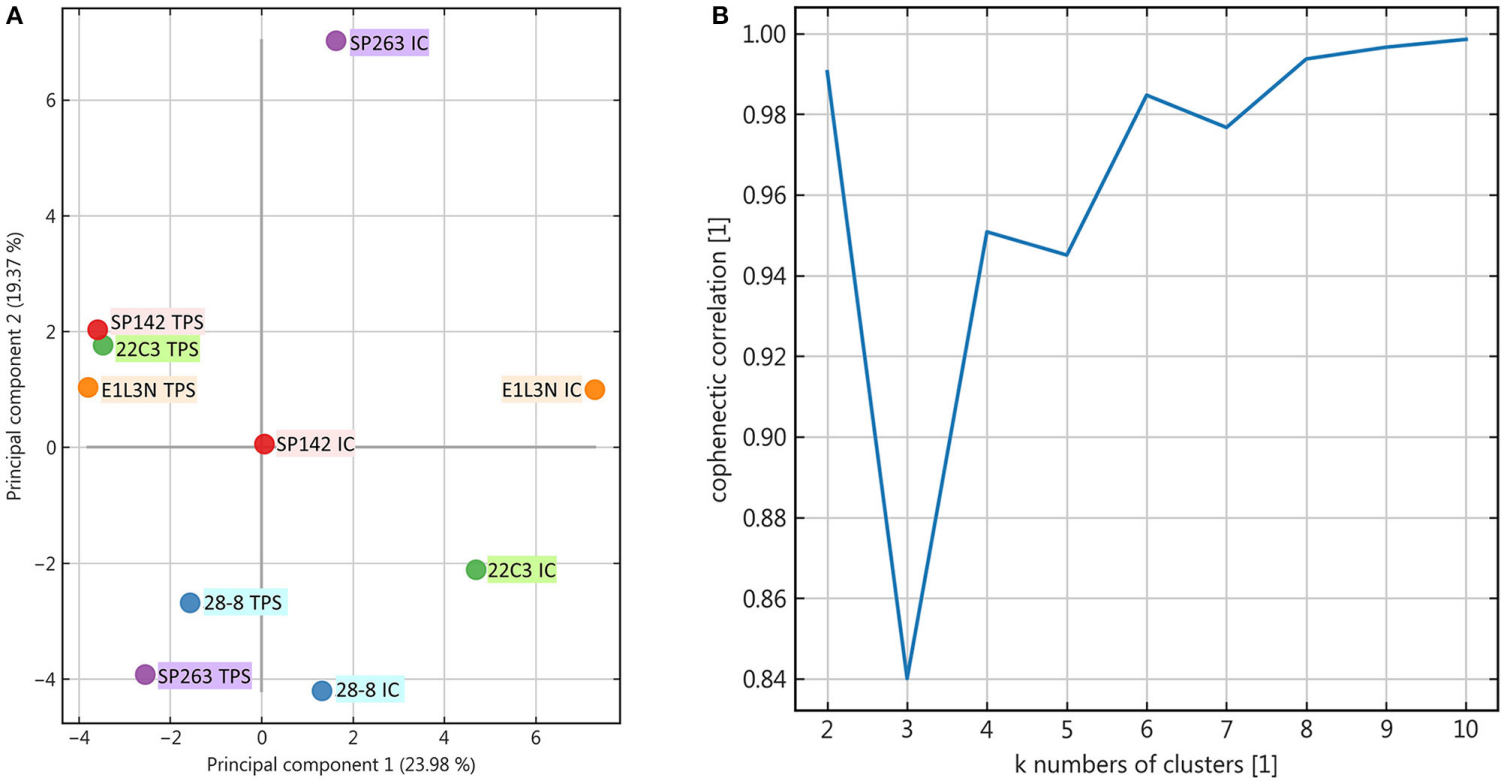
Principal component analysis and rank estimate for non-negative matrix factorization (NMF) consensus clustering. **(A)**: Principal components were calculated for the dataset and the individual clones visualized two-dimensionally according to their relative correlation with the two main components; **(B)**: In NMF the cophenetic correlation was calculated to find the number of clusters that reduced dimensionality best.

**Figure 5:**
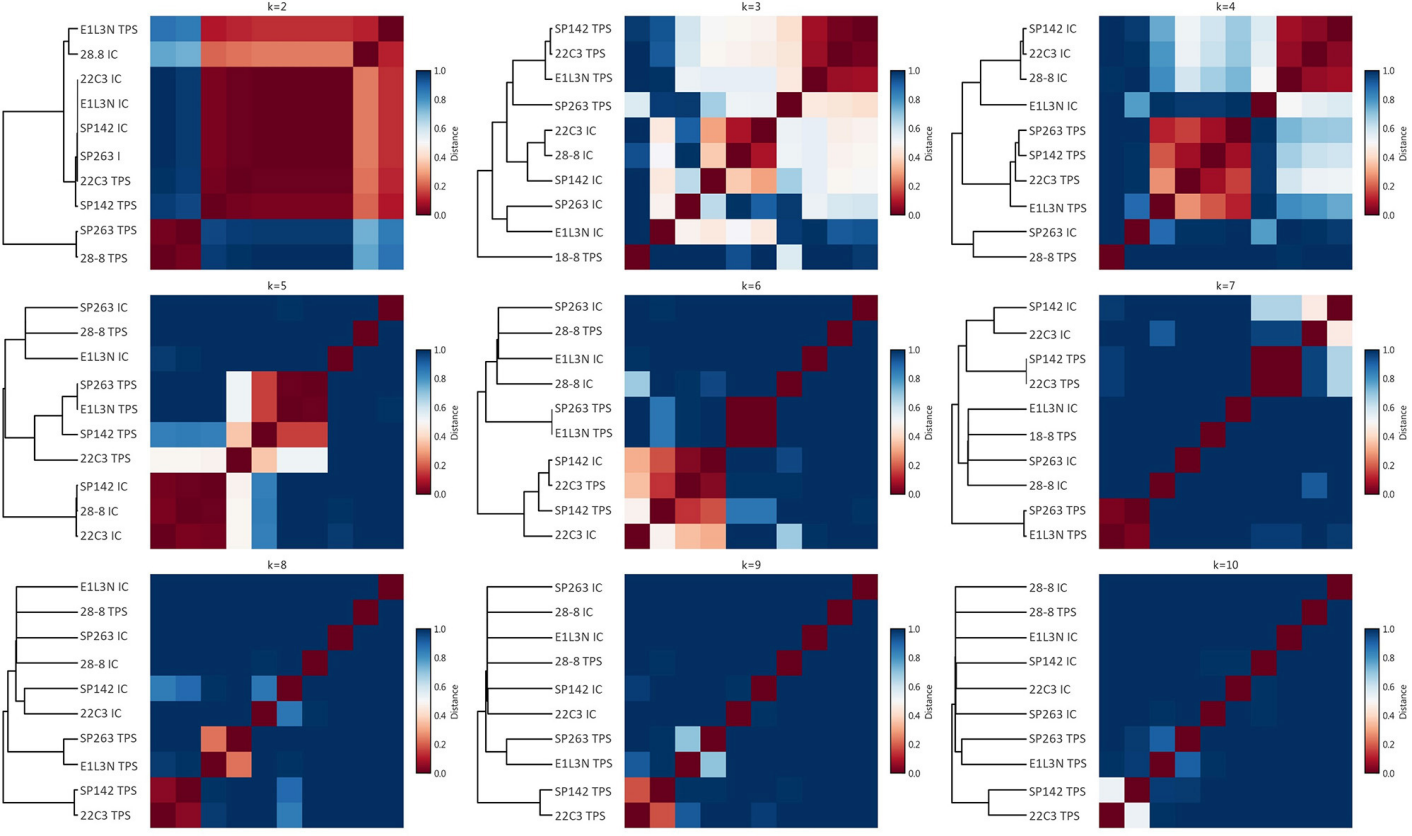
NMF consensus clustering. For varying numbers of clusters (ranks; subplots), NMF consensus clustering was performed; for each rank, the consensus matrix as a measure of cluster stability is shown as a heat plot with red indicating perfect consensus for a given pair of clones across stochastic runs (the clones end up in the same cluster all the time); the dendrogram on the left indicates relative cluster similarity by the respective lengths of the horizontal lines.

Apart from this qualitative assessment, the identification of underlying staining patterns was approached by principal component analysis (PCA) and non-negative matrix factorization-based consensus clustering (NMF). While PCA showed similarity between the tumor stainings of SP142, 22C3, and E1L3N (Figure 4A), no stable and relevant reduction of dimensionality could be observed by NMF clustering (Figure 5) indicated by maximum cophenetic correlation for k = 10 clusters (with *n* = 10 input samples (clones); Figure 4B). Some co-clustering of SP142/22C3 tumor staining was observed as well as less stable co-clustering of SP263 and E1L3N tumor staining. SP263 and 28-8 co-clustered relatively strongly for k = 2 clusters, mirrored by similar correlations in PCA and similar quantitative results of the staining raw data. However, the cluster did not prove to be stable in higher ranks (compare to quick drop in cophenetic correlation, Figure 4B).

## Discussion

The introduction of immune checkpoint inhibitors in cancer therapy considerably improved survival in several entities, e.g., melanoma and lung cancer (23, 24). For HNSCC, survival improved compared to the previous standard of care. However, the high hopes remained hitherto unfulfilled in large part. While overall survival for recurrent disease is still significantly increased in comparison with previous therapy regimens, the effect size seems to be smaller than in melanoma (8, 25). Adverse effects were significantly less severe than with chemotherapy. No more grade IV/V side effects were observed than with the comparator. Still, checkpoint inhibitors do offer side effects, some of which can be quite severe (e.g., pneumonitis, myocarditis). Against this background accurate checkpoint inhibitor response prediction in HNSCC patients is important.

Our data show a marked variation of staining results based on the diagnostic antibody used. Both descriptive analyses such as the basic share of positive cells as well as more comprehensive statistical approaches reveal only weak staining similarity, mostly between SP263 and 28-8 tumor staining. Taken altogether this translates into considerably different shares of patients being identified as eligible for second-line monotherapy.

Several harmonization studies for the diagnostic detection of PD-L1 expression were performed in different solid cancer entities with most studies focusing on non-small cell lung cancer (12, 14, 26–30). The results of the study presented here are only partially mirrored by investigations in other tumor entities. In two large studies of PD-L1 staining in lung cancer, three out of four assays showed interchangeable results (11, 31). In urothelial carcinoma, the different antibodies directed against PD-L1 showed different staining positivities but variance was still confined to an acceptable level. Here, too, three out of four assays were virtually interchangeable (32). In the above-mentioned studies, SP142 stained fewer samples, which was also observed in our data with SP142 identifying the fewest patients to be eligible for Pembrolizumab with CPS ≥ 1. In our study, the 22C3 clone showed CPS> = 1 in surprisingly much <85% reported in the KEYNOTE-048 (8) study. This may be due to tumor heterogeneity, selection bias, different pre-analytics, or staining platform protocols. Whatever the reason, this stark contrast further encourages to be cautions upon PD-L1 scoring results. Other comparative studies in non-small cell lung cancer found a high concordance in tumor cell scores for PD-L1 but apoor concordance in immune cells. Similarly, our results demonstrate slightly better concordance in tumor cell scoring compared to immune cells, which might affect CPS variance overproportionally. Overall comparability of scoring performance, however, was much lower in our data regardless of cell type than described in other entities.

The deviation of our results may in part root in the approach taken: To stay as close to real-world routine diagnostics as possible we did not average the results by multiple reviewing pathologists. The pronounced staining heterogeneity nonetheless suggests further systemic differences compared to other tumor entities. In this context, a potentially relevant aspect has been addressed by Lee et al. showing differences in the binding of Dako 28-8 before and after deglycosylation of FFPE samples (33). They concluded that the binding of a diagnostic antibody directed against PD-L1 is affected by the glycosylation state of the PD-L1 molecule itself. Since every antibody binds to a different epitope of the PD-L1 molecule, binding can be differentially affected by the glycosylation status of PD-L1 in each sample. Different levels of the inter-tumor variance of glycosylation patterns in HNSCC and other tumors might explain our observations—at least for the diagnostic antibodies that bind to the N-terminal extracellular domain [28-8 and 22C3; (34)].

Apart from discussing fixed diagnostic/therapeutic antibody pairs as companion diagnostic assays, it might be necessary to take a closer look at preanalytical optimization. Different glycosylation patterns in turn might not only be relevant in blurring formal eligibility criteria but also in better prediction of actual tumor biological behavior (33, 35). The different diagnostic scores in use add further complexity but only the CPS is currently applied clinically in HNSCC. We therefore restricted our primary comparisons to the CPS, with similar results for the TPS (Supplementary Figure 1). Given our present data, CPS determination in HNSCC should be interpreted with caution for therapeutic decisions. As CPS is crucial for therapeutic decision making a conceivable solution may be to establish an algorithm of testing various PD-L1 clones in succession to determine the CPS. In a back-to-back testing sequence, different PD-L1 clones could compensate for each other's “blind spots.”

## Data Availability Statement

The raw data supporting the conclusions of this article will be made available by the authors, without undue reservation.

## Ethics Statement

The studies involving human participants were reviewed and approved by University of Luebeck Ethics Committee (Ethikkommission der Universität zu Lübeck), Universität zu Lübeck, Ratzeburger Allee 160, 23538 Lübeck/Germany. Written informed consent for participation was not required for this study in accordance with the national legislation and the institutional requirements.

## Author Contributions

SP designed the study. Conceptualization by SP and SL. TMAs were constructed by JR-I, CI, and PK. RK, LK, CI, and CW maintained the cohort. JR-I, AO, and FD read the IHC slides and scored the stains. Statistical analyses were performed by FD supported by MR. BW, K-LB, and DR provided anonymized clinical data. JR-I, CI, FD, SL, and SP wrote the manuscript. Artwork was created by JR-I and F-OP. Graphs were created by FD. All authors contributed to the article and approved the submitted version.

## Conflict of Interest

The authors declare that the research was conducted in the absence of any commercial or financial relationships that could be construed as a potential conflict of interest.

## Supplementary Material

Supplementary Figure 1 Positive control for PD-L1 immunohistochemistry. Tonsil tissue, from top to bottom: 22C3, 28-8, E1L3N, SP142, and SP263. Original magnification x100. Inlay magnification x400.

Supplementary Figure 2 Clinical eligibility for alternative CPS and TPS cutoffs. **(A)** CPS 1 eligibility. **(B)** CPS 20 eligibility. **(C)** CPD50 eligibility. **(D)** TPS 50 eligibility.

Supplementary Table 1 Patient Data.
