# Supplementary figures and images for: Performance of Different Diagnostic PD-L1 Clones in Head and Neck Squamous Cell Carcinoma
Source: Front Med (Lausanne). 2021 Apr 27;8:640515. doi: 10.3389/fmed.2021.640515 (PMC8110724; doi:10.3389/fmed.2021.640515)

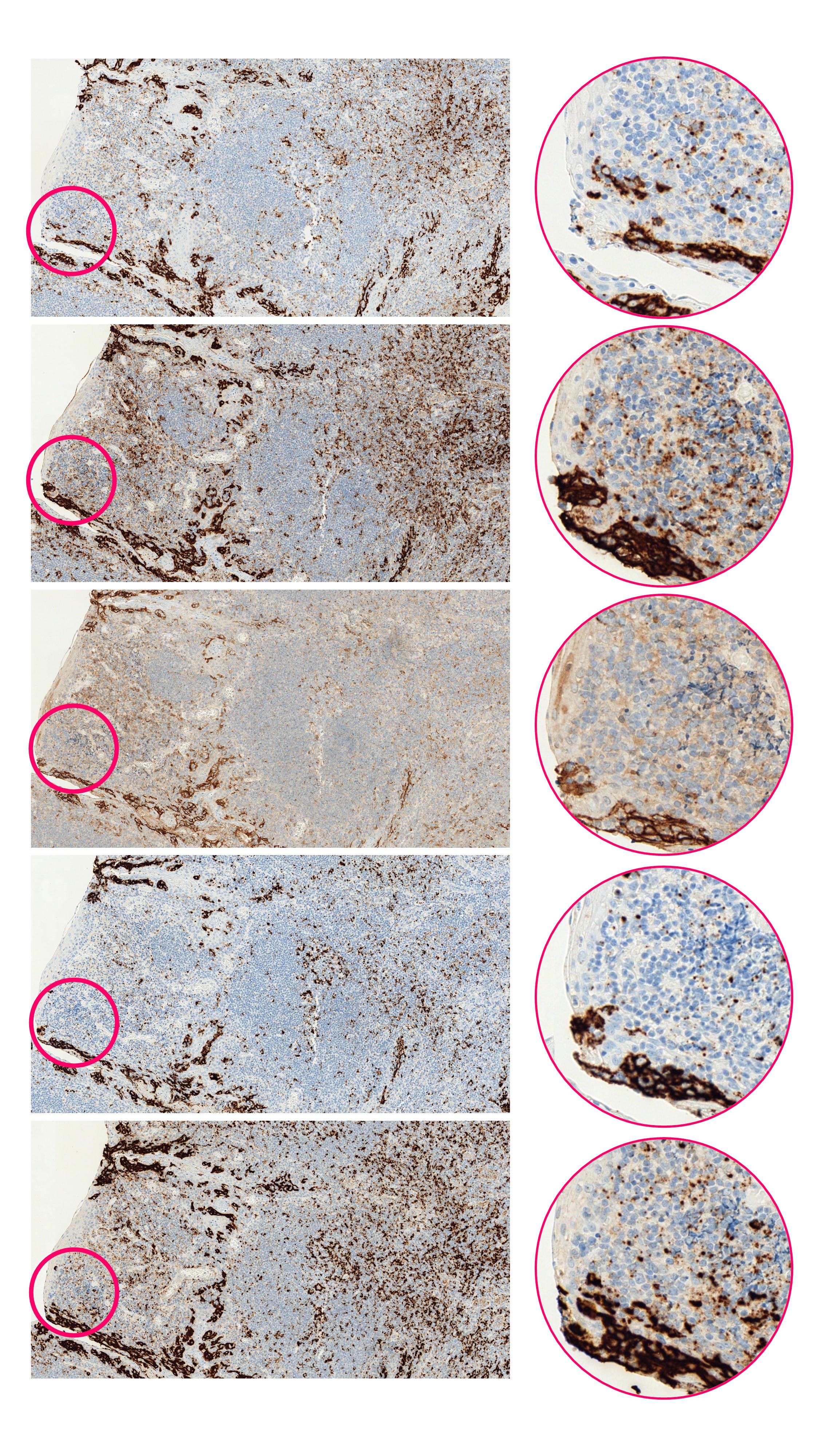

Supplement: Supplementary Figure 1 — Positive control for PD-L1 immunohistochemistry. Tonsil tissue, from top to bottom: 22C3, 28-8, E1L3N, SP142, and SP263. Original magnification x100. Inlay magnification x400. [file Image_1.JPEG]

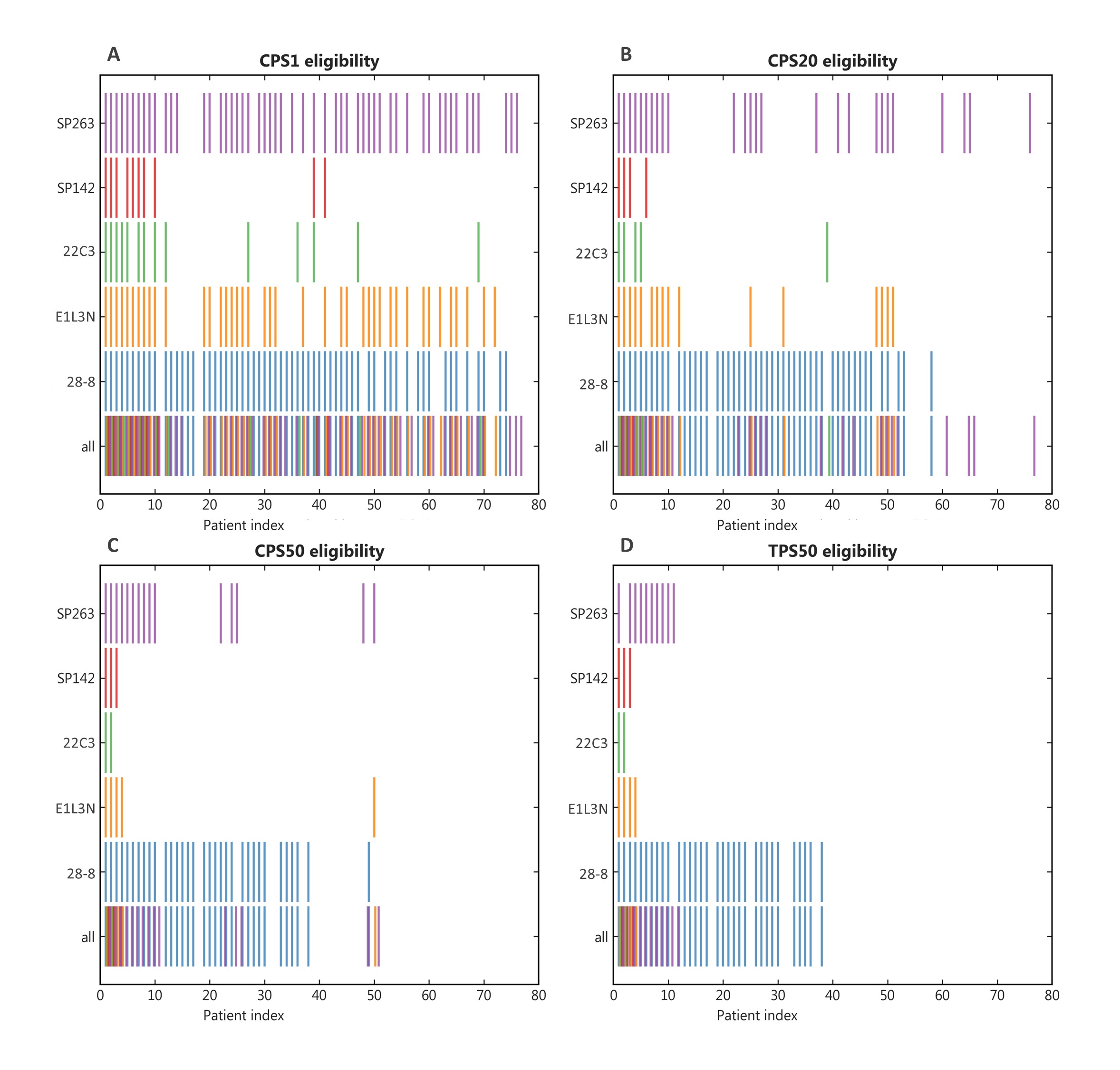

Supplement: Supplementary Figure 2 — Clinical eligibility for alternative CPS and TPS cutoffs. (A) CPS 1 eligibility. (B) CPS 20 eligibility. (C) CPD50 eligibility. (D) TPS 50 eligibility. [file Image_2.JPEG]
