# Supplementary material for: Performance of Different Diagnostic PD-L1 Clones in Head and Neck Squamous Cell Carcinoma
Source: Front Med (Lausanne). 2021 Apr 27;8:640515. doi: 10.3389/fmed.2021.640515 (PMC8110724; doi:10.3389/fmed.2021.640515)
Supplement: Supplementary Table 1 — Patient Data. [file Table_1.DOCX]

**Supplementary Material 1: Patient Data**

|  | **Age (yrs)** | **Sex** | **Localisation** | **T stage** | **N stage** | **UICC stage** | **Nicotine (packyears)** | **Alcohol abuse** | **p16 status** | **Immune Cell Pattern** | **Distant metastasis** | **Recurrent disease** |
| --- | --- | --- | --- | --- | --- | --- | --- | --- | --- | --- | --- | --- |
|  | 45 | male | oropharynx | T3 | N0 | UICC III | 30 | yes | negative | n/a | no | yes |
|  | 65 | female | oropharynx | T4 | N1 | UICC IV | 90 | yes | negative | n/a | no | yes |
|  | 80 | male | oral cavity | T2 | N0 | UICC II | 80 | no | negative | excluded | no | yes |
|  | 54 | male | oropharynx | T1 | N2 | UICC IV | 40 | yes | negative | excluded | yes | yes |
|  | 66 | male | oropharynx | T1 | N2 | UICC II | n/a | yes | negative | excluded | no | yes |
|  | 68 | male | larynx | T2 | N2 | UICC IV | 30 | no | negative | excluded | no | yes |
|  | 71 | male | larynx | T3 | N0 | UICC III | 50 | no | positive | cold | no | yes |
|  | 52 | male | larynx | T1 | N0 | UICC I | 80 | no | negative | excluded | yes | yes |
|  | 50 | male | larynx | T2 | N0 | UICC II | 35 | yes | negative | excluded | no | yes |
|  | 50 | female | oral cavity | T3 | N0 | UICC III | 40 | no | negative | hot | no | yes |
|  | 62 | male | larynx | T2 | N0 | UICC II | 15 | no | negative | excluded | no | yes |
|  | 76 | male | oropharynx | T2 | N1 | UICC I | 30 | yes | positive | excluded | no | yes |
|  | 74 | female | larynx | T3 | N1 | UICC III | 50 | no | negative | excluded | no | yes |
|  | 70 | male | oropharynx | T1 | N0 | UICC I | 30 | yes | positive | cold | no | yes |
|  | 54 | male | oropharynx | T4 | N2 | UICC IV | 40 | yes | positive | n/a | no | yes |
|  | 69 | male | hypopharynx | T3 | N0 | UICC III | n/a | n/a | negative | excluded | no | yes |
|  | 47 | male | oral cavity | T3 | N2 | UICC IV | 30 | yes | negative | excluded | no | yes |
|  | 58 | male | larynx | T2 | N0 | UICC II | 80 | yes | negative | excluded | no | yes |
|  | 82 | male | oral cavity | T1 | N0 | UICC I | n/a | n/a | negative | excluded | no | yes |
|  | 55 | male | oral cavity | T2 | N0 | UICC II | 46 | yes | negative | excluded | yes | yes |
|  | 57 | male | oral cavity | T3 | N2 | UICC IV | 30 | yes | negative | excluded | no | yes |
|  | 59 | female | oral cavity | T2 | N0 | UICC II | 0 | no | negative | excluded | no | yes |
|  | 40 | male | oropharynx | T2 | N3 | UICC IV | 20 | yes | negative | excluded | yes | yes |
|  | 70 | male | larynx | T2 | N0 | UICC II | 50 | no | negative | cold | yes | yes |
|  | 63 | female | oropharynx | T2 | N2 | UICC IV | 40 | yes | negative | n/a | no | yes |
|  | 38 | female | oral cavity | T3 | N0 | UICC III | 0 | no | positive | excluded | no | yes |
|  | 47 | female | hypopharynx | T4 | N2 | UICC IV | 33 | yes | negative | n/a | no | yes |
|  | 55 | male | larynx | T2 | N3 | UICC IV | n/a | no | negative | hot | no | yes |
|  | 60 | male | larynx | T3 | N0 | UICC III | 35 | no | negative | n/a | no | yes |
|  | 54 | male | hypopharynx | T3 | N2 | UICC IV | 30 | yes | negative | n/a | no | yes |
|  | 74 | female | larynx | T3 | N0 | UICC III | 0 | no | negative | hot | no | yes |
|  | 71 | male | larynx | T1 | N0 | UICC I | 50 | no | negative | excluded | no | yes |
|  | 51 | male | larynx | T1 | N0 | UICC I | 0 | no | positive | n/a | no | yes |
|  | 68 | female | oral cavity | T1 | N0 | UICC I | 10 | no | negative | excluded | no | yes |
|  | 53 | male | oral cavity | T3 | N0 | UICC III | 35 | no | negative | hot | no | yes |
|  | 85 | female | oral cavity | T1 | N0 | UICC I | 66 | no | negative | cold | no | yes |
|  | 78 | male | oral cavity | T3 | N2 | UICC IV | 35 | no | negative | excluded | no | yes |
|  | 69 | male | larynx | T2 | N1 | UICC III | 35 | no | negative | excluded | no | yes |
|  | 51 | male | oral cavity | T1 | N0 | UICC I | 35 | yes | negative | hot | no | yes |
|  | 87 | male | other | T2 | N2 | UICC I | n/a | n/a | negative | n/a | no | yes |
|  | 47 | male | oral cavity | T2 | N0 | UICC II | 17 | yes | negative | excluded | no | yes |
|  | 75 | male | larynx | T3 | N0 | UICC IV | 40 | no | negative | cold | yes | yes |
|  | 45 | male | hypopharynx | T3 | N2 | UICC IV | 26 | yes | negative | hot | no | yes |
|  | 49 | male | hypopharynx | T4 | n/a | UICC IV | 30 | yes | negative | excluded | yes | yes |
|  | 72 | male | larynx | T2 | N0 | UICC II | 50 | no | positive | excluded | no | yes |
|  | 53 | female | oropharynx | T3 | N2 | UICC IV | 10 | yes | negative | n/a | no | yes |
|  | 59 | male | oral cavity | T3 | N2 | UICC IV | 40 | no | negative | excluded | no | yes |
|  | 61 | male | oral cavity | T4 | N0 | UICC IV | 0 | no | negative | excluded | no | yes |
|  | 64 | female | oropharynx | T3 | N1 | UICC II | 15 | no | positive | excluded | no | yes |
|  | 71 | female | oral cavity | T2 | N2 | UICC IV | 50 | no | negative | excluded | no | yes |
|  | 58 | male | oral cavity | T1 | N0 | UICC I | n/a | no | negative | cold | no | yes |
|  | 82 | male | oropharynx | T4 | N2 | UICC IV | n/a | n/a | positive | n/a | no | yes |
|  | 53 | male | larynx | T3 | N0 | UICC III | 30 | no | negative | excluded | no | yes |
|  | 56 | male | hypopharynx | T4 | N3 | UICC IV | 37 | yes | negative | n/a | no | yes |
|  | 66 | male | oropharynx | T2 | N0 | UICC II | 45 | yes | negative | excluded | yes | no |
|  | 57 | male | larynx | T4 | N0 | UICC IV | n/a | yes | negative | excluded | yes | no |
|  | 48 | male | other | T3 | N2 | UICC IV | 90 | yes | negative | n/a | no | no |
|  | 62 | male | oropharynx | T3 | N3 | UICC IV | 42 | yes | positive | hot | no | no |
|  | 52 | male | other | T2 | N1 | UICC II | 30 | yes | positive | hot | yes | no |
|  | 50 | male | hypopharynx | T3 | N0 | UICC III | 45 | yes | negative | hot | no | no |
|  | 72 | male | larynx | T3 | N0 | UICC III | 40 | no | positive | cold | yes | no |
|  | 48 | male | oral cavity | T1 | N0 | UICC I | 45 | yes | negative | hot | no | no |
|  | 66 | male | oral cavity | T2 | N1 | UICC I | 60 | no | positive | excluded | yes | no |
|  | 52 | male | larynx | T4 | N1 | UICC IV | 35 | no | negative | cold | yes | no |
|  | 44 | male | oropharynx | T4 | N3 | UICC IV | 23 | yes | negative | n/a | yes | no |
|  | 65 | female | oropharynx | T1 | N0 | UICC I | 35 | no | negative | excluded | yes | no |
|  | 80 | male | larynx | T3 | N0 | UICC III | 50 | no | negative | excluded | no | no |
|  | 53 | male | oropharynx | T1 | N0 | UICC I | 35 | yes | negative | hot | yes | no |
|  | 74 | male | oral cavity | T4 | N2 | UICC IV | 0 | no | negative | hot | no | no |
|  | 60 | male | hypopharynx | T3 | N3 | UICC IV | n/a | n/a | negative | n/a | no | no |
|  | 59 | male | oral cavity | T1 | N0 | UICC I | 40 | no | negative | excluded | no | no |
|  | 60 | female | larynx | T2 | N0 | UICC II | 40 | no | negative | excluded | yes | no |
|  | 63 | male | larynx | T2 | N0 | UICC II | 50 | no | negative | excluded | no | no |
|  | 62 | female | oral cavity | T2 | N2 | UICC IV | 20 | no | negative | hot | no | no |
|  | 63 | male | hypopharynx | T4 | N3 | UICC IV | 45 | no | negative | hot | yes | no |
